# A Case of Mistaken Identity: Varicella Zoster and Monkeypox

**DOI:** 10.7759/cureus.41775

**Published:** 2023-07-12

**Authors:** Hamza Malick, Ronnie Youssef, Kirstin Altman

**Affiliations:** 1 Medical School, Texas A&M College of Medicine, Dallas, USA; 2 Dermatology, Baylor Scott & White Health, Temple, USA

**Keywords:** monkeypox virus rash, monkeypox virus, varicella-zoster virus infection, real-time pcr, varicella-zoster virus

## Abstract

Mpox, previously referred to as monkeypox, is a zoonotic virus originally endemic to West Africa which has recently garnered significant attention due to a global outbreak. It remains a challenging diagnosis due to varying clinical presentations and similarities with other infectious pathogens. While diligent monitoring of its prevalence remains crucial, clinicians should combat recency bias when forming differentials for viral illnesses with similar presentations. Here, we discuss the case of an immigrant child with self-reported vaccination history of Varicella Zoster who was diagnosed with Mpox in the emergency department but was subsequently found to have Varicella Zoster after further testing. To effectively manage outbreaks and provide optimal care, healthcare professionals should stay up to date on the latest advancements in diagnostic techniques and available interventions.

## Introduction

Mpox, previously referred to as monkeypox, is a zoonotic orthopoxvirus originally endemic to West Africa [1]. While the reservoirs of the virus include monkeys, rodents, and prairie dogs, human-to-human transmission can occur through respiratory droplets, percutaneous exposure of body fluids, and contact with infected skin lesions [1]. Recently, Mpox has garnered significant media attention, primarily due to limited familiarity with the disease and its atypical presentation. The rapid international outbreaks of monkeypox that followed closely after the SARS-CoV-2 (COVID-19) pandemic have fueled the concern surrounding the disease [2]. The convergence of these factors has contributed to heightened awareness within medical and civil communities and has placed a greater emphasis on the management of this disease. While it is important to maintain a high degree of caution for this resurging infectious pathogen, clinicians should combat recency bias when forming differentials for other viral illnesses that may present similarly.

## Case presentation

**Figure 1:**
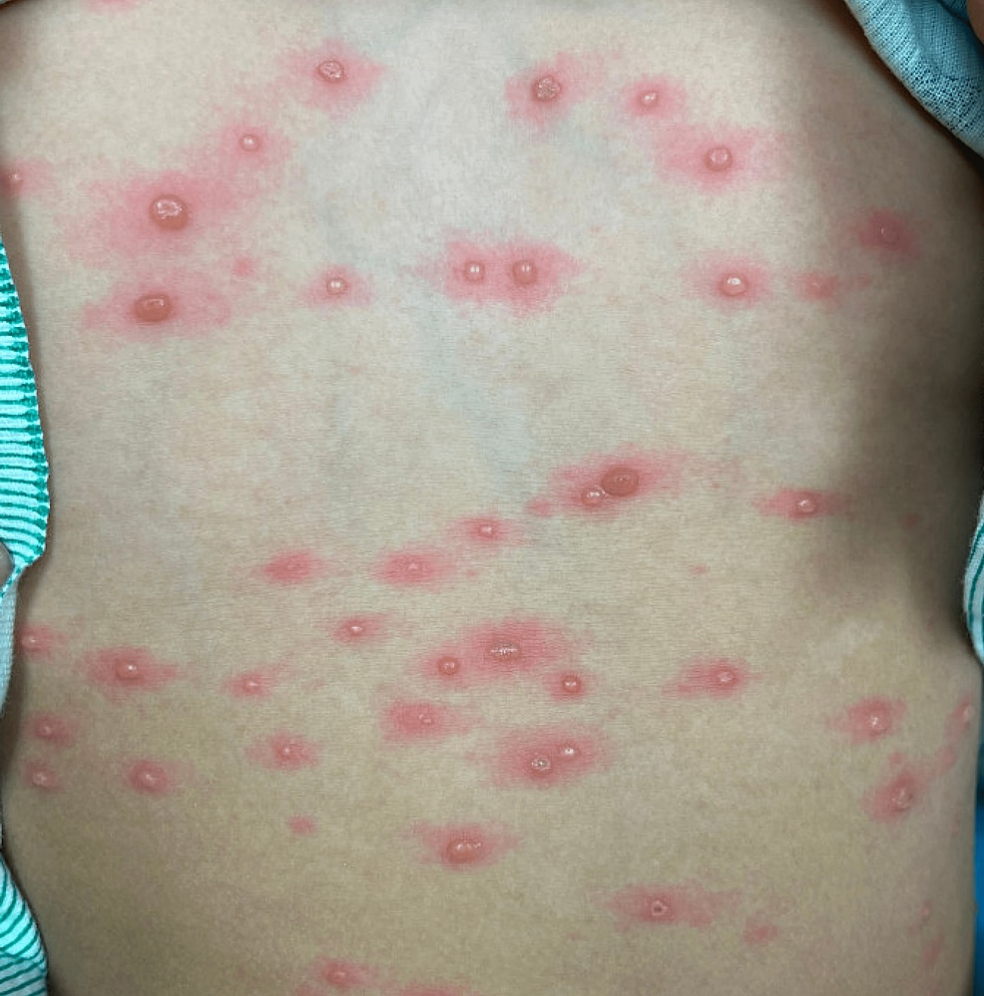
Tense umbilicated vesicles on the patient’s trunk.

An eight-year-old girl presented to the emergency department with a three-day history of a diffuse vesicular rash that started on her chest and quickly spread to her face, back, extremities, perineum, and palate. The patient had started exhibiting prodromal symptoms of congestion, rhinorrhea, and cough without fever four days before hospital admission. The patient denied any prior reactions, history of rashes, pertinent past medical history, or recent medication use. Her family further denied recent exposure to sick contacts, changes in topical hygiene products, or proximity to wildlife. No other family members reported similar symptoms or rashes. The patient had recently immigrated from Mexico six months prior and had a self-reported vaccination history without documentation. The patient had a reported history of Varicella-Zoster virus (VZV) infection and subsequent vaccination in childhood without accurate recall of the timing or dose of vaccinations and infectious timeline. On physical examination, the child appeared afebrile and exhibited tense umbilicated vesicles, all in the same stages of development, on an erythematous base affecting the face, trunk, perineum, and extremities (Figure 1).

Given the patient’s reported vaccination history and prior infection with VZV, the recurrence of the viral pathogen was considered atypical. Furthermore, due to the community spread of Mpox with rising cases, suspicion remained high for this viral pathogen. While the patient was placed under appropriate isolation precautions, a polymerase chain reaction (PCR) test of a lesion was ordered for orthopoxvirus, which yielded a negative result. Subsequently, a VZV PCR test was conducted due to the persistence of clinical symptoms. The results were positive, and a VZV antibody screen further confirmed a lack of immunity to the virus. The patient was initiated on oral acyclovir for five days, resulting in the resolution of symptoms.

## Discussion

Mpox remains a challenging diagnosis due to varying clinical presentations and similarities with other infectious pathogens such as smallpox, varicella, herpes simplex virus, measles, molluscum contagiosum, syphilis, and rocky mountain spotted fever [1,3]. The presence of a vesiculopustular rash with centrifugal spread, as well as nonspecific findings such as headache, rhinorrhea, low-grade fever, and lethargy, have been increasingly associated with prodromal symptoms in Mpox patients [2,3]. As the global outbreak of this virus continues, clinicians have studied the use of PCR for accurate and timely diagnosis of Mpox [4,5]. These studies have revealed that while a skin swab often yields positive results for Mpox DNA, it is recommended that medical practitioners obtain swabs from at least two different sites, including mucosal regions, to further enhance diagnostic accuracy [1,5].

Although no specific treatment for Mpox has been approved, tecovirimat has recently been established by the Centers for Disease Control and Prevention as a primary treatment option [6]. Other antiviral medications, including brincidofovir and acyclovir, have also been considered for many patients, particularly those at risk of prolonged or severe complications [4,6]. While VZV in immunocompetent adolescents is often limited in severity and treated with supportive therapies, several studies have discussed the benefits of early intervention with oral acyclovir to reduce the severity, systemic symptoms, and duration of the disease [7]. Similarly, adults diagnosed with VZV are frequently prescribed oral acyclovir at the onset of the disease to mitigate its course.

As immigrant and refugee populations continue to rise, it becomes increasingly important to consider a thorough differential diagnosis and understand the socioeconomic factors affecting special populations. The presented case is an example of an immigrant child with an inconsistent immunization history, which played a defining role in treatment. Further epidemiological studies are also needed to investigate the use of medication-based interventions and their impact on disease transmission, as well as treatment strategies for unvaccinated or immunocompromised populations.

## Conclusions

Mpox can present with a myriad of symptoms and has increasingly been linked with viral prodromes. As Mpox continues to remain a global phenomenon, diligent monitoring and understanding of its prevalence and diagnostic guidelines are crucial. It is imperative to note that the symptoms and cutaneous findings observed in this case were not specific to Mpox and could be attributed to VZV and similar viral illnesses. This case highlights the importance of comprehensive testing and considering a wide range of possibilities when diagnosing infectious pathologies. Therefore, in the case of this patient, it was crucial to rely not only on clinical judgment based on a thorough history but also to consider a broader differential, which included more common viral pathogens, particularly when ordering diagnostic laboratory tests. Her recent immigration history and lack of immunization documentation should have been considered when formulating appropriate management and developing a comprehensive differential.
